# Supplementary material for: Identification of photoperiod-induced specific miRNAs in the adrenal glands of Sunite sheep (Ovis aries)
Source: Front Vet Sci. 2022 Jul 22;9:888207. doi: 10.3389/fvets.2022.888207 (PMC9354845; doi:10.3389/fvets.2022.888207)
Supplement: Supplementary file 1 [file Data_Sheet_1.ZIP › Supplementary file/miRDeep2 file.zip!/miRDeep2 file/Novel_Structure/SPLP42A2/Novel_707.pdf]

Provisional ID	:	NC_019480.2_38665
Score total	:	0.3
Score for star read(s)	:	-1.3
Score for read counts	:	0
Score for mfe	:	0
Score for randfold	:	1.6
Score for cons. seed	:	
Total read count	:	60
Mature read count	:	55
Loop read count	:	0
Star read count	:	5

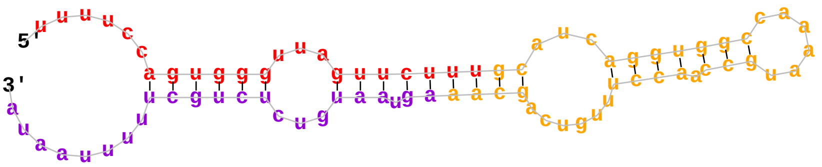

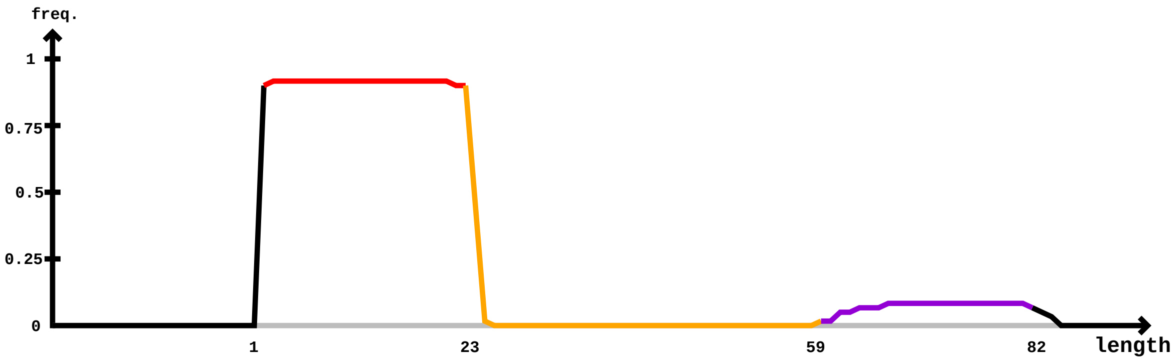

Mature Star

```
5'- ucuuuccuaguaucaggguc uuuuccaguggguuaguucuuu gcaucagguggccaaaaugccaaccuuugucagcaa aguaaugucucugcuuuuuaaua ugcugucuagg -3'    obs
```

```
5'- ucuuuccuaguaucagggucuuuuccaguggguuaguucuuugcaucagguggccaaaaugccaaccuuugucagcaaaguaaugucucugcuuuuuaauaugcugucuagg   -3'   obs
    ucuuuccuaguaucagggucuuuuccaguggguuaguucuuugcaucagguggccaaaaugccaaccuuugucagcaaaguaaugucucugcuuuuuaauaugcugucuagg         exp
    .....(((((...(((.((.((....((((((...(((((((((...(((((((......))).))))......)))))).)))...))))))....)).)).))).)))))   reads   mm   sample
    ....................uuuuccagugAguuaguucu........................................................................   1       1    seq
    ....................uuuuccagugAguuaguucuuu......................................................................   45      1    seq
    ....................uuuuccaguggguuaguucuuu......................................................................   1       0    seq
    ....................uuuuccagugAguuaguucuuug.....................................................................   6       1    seq
    ....................uuuuccagugAguuaguucuuugc....................................................................   1       1    seq
    .....................uuuccagugAguuaguucuuug.....................................................................   1       1    seq
    ..............................................................................aguaaugucucugcuuuuuaaua...........   1       0    seq
    ................................................................................uaaugucucugcuuuuuaau............   1       0    seq
    ................................................................................uaaugucucugcuuuuuaauaGg.........   1       1    seq
    ..................................................................................augucucugcuuuuuaauau..........   1       0    seq
    .....................................................................................ucucugAuuuuuaauaug.........   1       1    seq
```
